# Thymoma-associated stiff-person syndrome successfully treated with extended thymectomy: a case report

**DOI:** 10.1186/s44215-026-00264-3

**Published:** 2026-06-19

**Authors:** Kie Maita, Tomoki Higeta, Hajime Watanabe, Takayuki Nakano, Kentaro Tokuoka, Tomoki Nakagawa, Shigeru Nogawa, Shunsuke Yamada, Ryota Masuda

**Affiliations:** 1 Division of General Thoracic Surgery, Tokai University Hachioji Hospital, Hachioji, Tokyo, Japan; 2 Department of Neurology, Tokai University Hachioji Hospital, Tokai University School of Medicine, Tokyo, Japan; 3 Department of Surgery, Division of Thoracic Surgery, Tokai University School of Medicine, Isehara, Kanagawa Japan

**Keywords:** Stiff-person syndrome, Thymoma, Extended thymectomy, Paraneoplastic neurological syndrome

## Abstract

**Background:**

Stiff-person syndrome (SPS) is a rare autoimmune neurological disorder characterized by painful muscle stiffness and spasms. Paraneoplastic SPS associated with thymoma is rare. Here, we describe a rare case in which neurological symptoms improved after extended thymectomy.

**Case presentation:**

A 60-year-old woman presented with progressive painful muscle spasms and gait disturbances affecting the lower extremities. Neurological imaging revealed no structural abnormalities; however, both serum and cerebrospinal fluid were positive for anti-glutamic acid decarboxylase (GAD) antibodies, leading to a diagnosis of SPS. Despite receiving immunotherapy and symptomatic treatment, the patient’s neurological symptoms persisted. Contrast-enhanced chest computed tomography revealed a small anterior mediastinal tumor suspected to be a thymoma. Subsequently, bilateral thoracoscopic extended thymectomy was performed. Histopathological examination confirmed a WHO type B1 thymoma (Masaoka stage I), and her neurological symptoms gradually improved postoperatively. Two years after surgery, she was able to walk independently and climb stairs without assistance.

**Conclusion:**

This case suggests that extended thymectomy may contribute to sustained neurological and functional improvement in selected patients with thymoma-associated SPS, even in the absence of normalization of the anti-GAD antibody titer.

## Background

Stiff-person syndrome (SPS) is a rare autoimmune neurological disorder characterized by progressive muscle stiffness and painful spasms that predominantly involve the axial muscles, resulting in gait disturbances. This condition was first described by Moersch and Woltman in 1956 as stiff-man syndrome [1]. The estimated incidence is approximately one per million individuals, with a female predominance and a typical onset between 30 and 60 years of age. Approximately 5% of cases are considered paraneoplastic, most associated with breast cancer, whereas an association with thymoma is rare [2, 3].

Here, we report a rare case of thymoma-associated SPS in which neurological symptoms improved after extended thymectomy.

## Case presentation

A 60-year-old woman with no significant medical or family history noticed discomfort in the sole of her right foot during yoga practice, followed by progressive rigidity of the right ankle. Two weeks later, painful muscle stiffness developed in both lower extremities, resulting in difficulty standing and walking. The patient was admitted to the Department of Neurology at our institution to undergo further evaluation.

Neurological examination revealed bilateral spasticity, predominantly on the right side, accompanied by hyperactive deep tendon reflexes and a positive Babinski sign. Laboratory testing demonstrated markedly elevated serum anti-glutamic acid decarboxylase (GAD) antibody levels (132,000 U/mL), and cerebrospinal fluid analysis showed elevated anti-GAD antibody levels (3,520 U/mL) without pleocytosis. Serum anti-acetylcholine receptor (AChR) antibodies were weakly positive; however, no clinical manifestations suggestive of Myasthenia Gravis (MG) was observed. No pleocytosis or decreased glucose levels were observed in the cerebrospinal fluid, and anti-amphiphysin antibodies were negative. Magnetic resonance imaging of the brain and spine revealed no abnormalities.

**Fig. 1:**
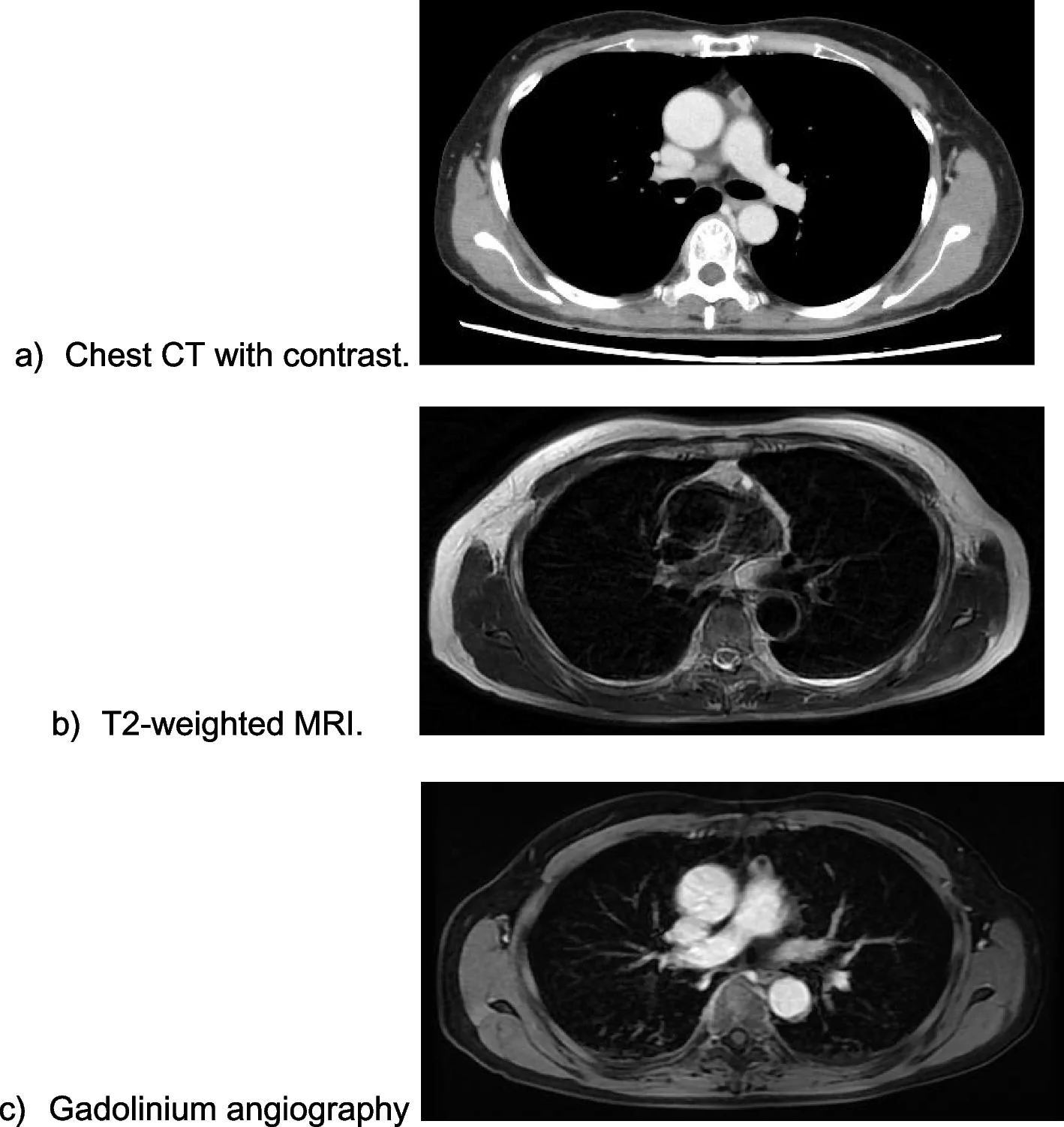
Chest computed tomography (CT) and magnetic resonance imaging (MRI) findings. An anterior mediastinal tumor measuring 13 mm in maximal diameter demonstrates partial contrast enhancement on contrast-enhanced CT (**a**). On T2-weighted MRI, the tumor exhibits areas of high signal intensity (**b**) with mild peripheral enhancement on gadolinium-enhanced images (**c**)

Contrast-enhanced chest computed tomography identified a 13 × 10 × 2 mm anterior mediastinal nodule with heterogeneous enhancement. Chest magnetic resonance imaging revealed focal high-intensity areas on T2-weighted imaging with mild peripheral enhancement (Fig. 1). Based on these findings, paraneoplastic SPS associated with thymoma was suspected.

Treatment with high-dose intravenous methylprednisolone, as well as intravenous immunoglobulin (IVIG), baclofen, and diazepam, led to rapid improvement in lower limb pain. Lower limb spasticity gradually improved but persisted, and the patient still required assistance to stand and was unable to walk independently. Following a multidisciplinary discussion, bilateral thoracoscopic extended thymectomy was performed after tapering the prednisolone dose to 10 mg/day.

### Surgical findings

Bilateral thoracoscopic extended thymectomy was performed without complications. The procedure was initiated on the right side to facilitate clear identification of the left brachiocephalic vein. The tumor showed no invasion into the surrounding tissues, allowing complete resection. The operative time was 3 h and 1 min, and the estimated blood loss was 34 ml.

### Pathological findings

**Fig. 2:**
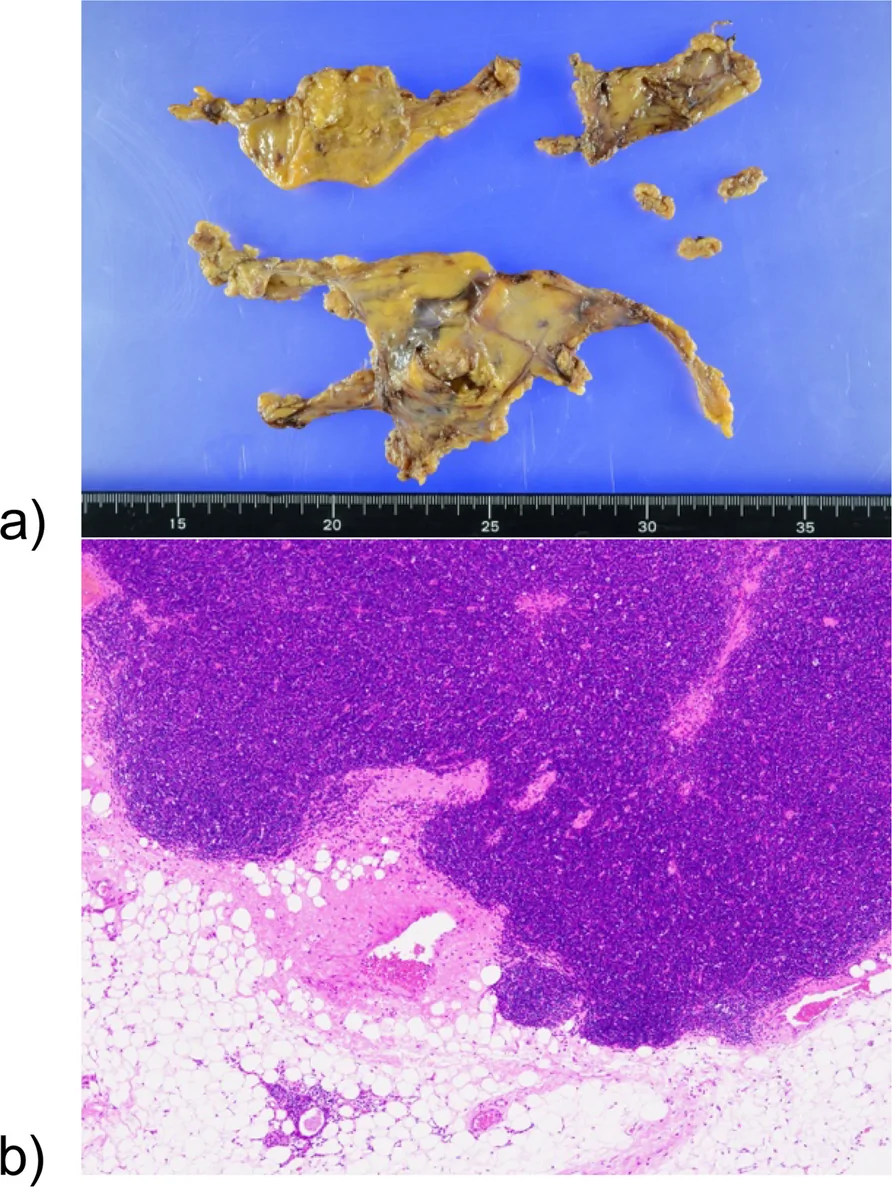
Findings of the resected tumor. Macroscopically, the resected tumor was grayish-white and measured approximately 20 mm in diameter (**A**). Microscopic examination with hematoxylin and eosin staining at low magnification (**b**; × 20) reveals a WHO type B1 thymoma without capsular invasion

Histopathological examination revealed a nodular lesion predominantly composed of lymphocytes within mature adipose tissue. Immunohistochemical staining revealed a reticular network of keratin-positive epithelial cells (AE1/AE3) and immature TdT-positive lymphocytes, consistent with thymoma (Fig. 2). The final diagnosis was a WHO type B1 thymoma with Masaoka stage I and complete resection (R0).

### Postoperative course

The patient’s postoperative course was uneventful. The patient continued intensive rehabilitation and showed gradual improvement in mobility. She was discharged on postoperative day 32 and ambulated with a cane. However, after discharge, she experienced temporary worsening of symptoms, including increased spasticity and a higher stiffness index, requiring readmission. Her symptoms improved after treatment with methylprednisolone.

**Fig. 3:**
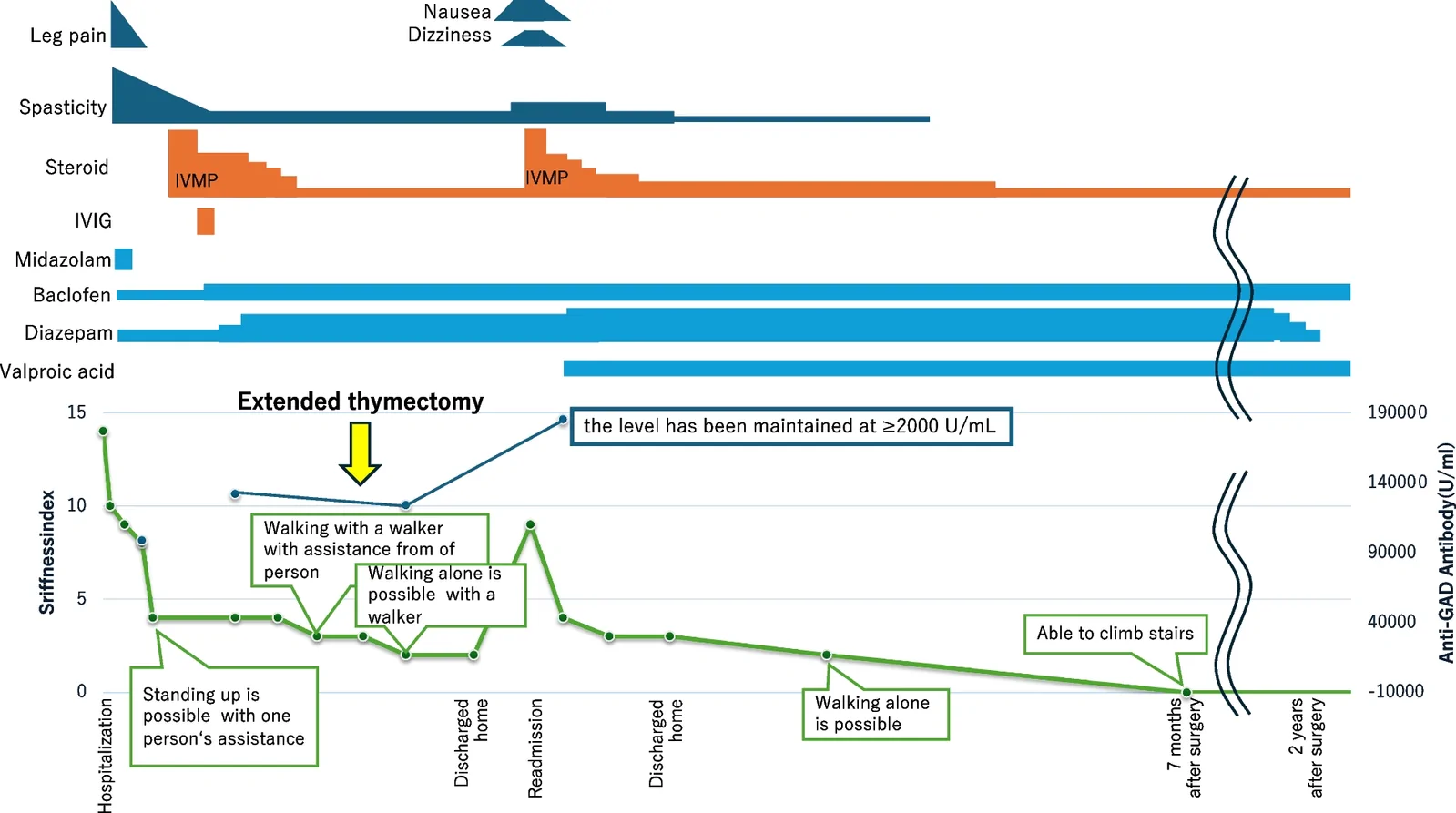
Clinical course and treatment timeline. The upper panel shows symptoms and treatments over time. IVMP, intravenous methylprednisolone; IVIG, intravenous immunoglobulin. The lower panel shows changes in the stiffness index and functional status. Wavy lines indicate a break in the time axis

Laboratory tests obtained before discharge showed a decrease in anti-GAD antibody levels to 124,000 U/mL in serum and 3,470 U/mL in cerebrospinal fluid, representing a downward trend compared with preoperative values, although both remained markedly elevated. Subsequently, serum anti-GAD antibody levels remained above 5,000 U/mL during follow-up. Two years after extended thymectomy, the patient was able to walk independently and climb stairs without assistance, and diazepam was discontinued (Fig. 3). She was maintained on prednisolone at 10 mg/day with stable neurological symptoms, and gradual tapering was considered.

## Discussion

SPS, a rare autoimmune neurological disorder characterized by impaired inhibitory γ-aminobutyric acid (GABA)ergic neurotransmission, is frequently associated with altered anti-GAD antibodies [4, 5]. Anti-GAD antibodies are detected in approximately 70–80% of patients with SPS [5], and associations with other autoimmune diseases, including type 1 diabetes mellitus and Hashimoto’s thyroiditis, have been reported [6]. Treatment includes symptomatic therapies such as benzodiazepines and baclofen, which enhance GABAergic neurotransmission, and immunomodulatory therapies, including IVIG, plasma exchange, corticosteroids, and rituximab [7].

Approximately 5% of SPS cases are paraneoplastic, most commonly associated with breast cancer, whereas thymoma-associated SPS is rare [2, 8]. Although thymoma-associated SPS is often grouped with paraneoplastic SPS, its immunopathological profile differs from classical anti-amphiphysin antibody-positive SPS associated with breast cancer or small-cell lung carcinoma. Thymoma-associated SPS commonly coexists with other autoimmune disorders and may present with anti-GAD antibody positivity, reflecting immune dysregulation related to thymic epithelial tumors. In the present case, weak anti-AChR antibody positivity without clinical MG, together with negative anti-amphiphysin antibodies, supported this thymoma-related autoimmune background.

The present case had a rapidly progressive course. Although diazepam and baclofen partially relieved symptoms, functional impairment persisted. Because surgical stimulation may exacerbate SPS and sensitivity to neuromuscular blocking agents may cause prolonged muscle relaxation or postoperative respiratory failure [9], perioperative stabilization was considered essential. Thymomas are generally slow-growing tumors, and neurological improvement after thymectomy may require weeks to months. Therefore, corticosteroids, plasmapheresis, and IVIG were administered preoperatively to stabilize SPS before surgery.

A minimally invasive approach was selected to reduce chest wall trauma and the risk of postoperative worsening of rigidity, while avoiding sternotomy-related complications in a patient receiving corticosteroids. Bilateral thoracoscopic extended thymectomy was performed after neurological stabilization and tapering prednisolone to 10 mg/day. This strategy aimed to maintain oncological radicality while minimizing perioperative stress and steroid-related complications, including impaired wound healing and infection.

To date, 16 surgically treated cases of thymoma-associated SPS have been reported (Table 1) [10–25]. Most patients were anti-GAD antibody-positive (15 of 16), and anti-AChR antibodies were present in 7 of 16. Coexisting autoimmune diseases were frequent, with MG being the most common; MG was present at diagnosis in three cases and developed postoperatively in two cases. Pure red cell aplasia and autoimmune thyroiditis were also reported.

**Table 1:** Overview of previously reported cases of thymoma-associated stiff-person syndrome treated surgically

No	Age	Sex	anti-GAD	anti-AChR	surgical procedure	WHO histology	response to surgery	worsening of SPS symptoms	Othoer immune complications	Author (Year)	Ref
1	55	M	-	-	Thx	B2	well	post ope	MG-post	Nicholas AP (1997)	[12]
2	40	F	+	-	Ext-Thx + RT	B1	well	-	-	Hagiwara H (2001)	[13]
3	45	M	+	N → P	Thx	NR	well	-	MG-post	Thomas S (2005)	[14]
4	57	F	+	-	R-ext- Thx	B1	well	post ope	-	Tanaka H (2005)	[11]
5	79	F	+	NR	Ext- Thx	AB	well	post ope	-	Iwata T (2006)	[15]
6	51	M	+	-	Thx	B1	well	-	-	Essalmi L (2007)	[16]
7	53	M	+	NR	Thx	NR	well	post ope	AITD-simul	Dupond JL (2009)	[17]
8	81	F	+	NR	Ext- Thx	B1	well	post ope/recurance	PRCA-post	Kobayashi R (2014)	[18]
9	72	F	+	+	Ext- Thx	B2	poor	post ope	MG-simul	Morise S (2017)	[19]
10	68	M	+	-	R-Thx	B2	well		-	Sonokawa (2019)	[23]
11	30	M	+	+	R- Thx	NR	well	post ope	MG-simul	Mehta MP (2020)	[20]
12	26	F	+	+	Ext- Thx	B3	well	-	-	Sasaki A (2021)	[21]
13	47	M	+	+	Thx(not ditailed)	B2	well	post ope	-	Perri M (2023)	[22]
14	57	M	+	-	R-ext- Thx	B1	well	-	-	Sumiya R (2024)	[24]
15	74	M	+	+	Robo-ext- Thx	B2	well	-	MG-simul	Okado S (2025)	[10]
16	65	F	+	+	VATS -ext- Thx	B1	well	post ope	-	present case	

*AITD* Autoimmune thyroid disease, *ext* extended, *VATS* video assisted thoracoscopic surgery, *N → P* normal → positive, *MG* myasthenia gravis, *NR* Not reported, *PRCA* Pure Red Cell Aplasia, *R* radical Robo, robotic, *Thx* Thymectomy

Surgical outcomes were generally favorable, with many patients showing neurological improvement. However, postoperative relapse or exacerbation occurred in nine patients (56%), including the present case. These findings suggest that surgery alone may not immediately stabilize SPS and that adjunctive immunotherapy is often required. Although minimally invasive approaches may reduce surgical stress, the limited number of thoracoscopic and robotic cases prevents firm conclusions regarding the influence of surgical approach on neurological outcomes. Available evidence suggests that postoperative exacerbation is more strongly related to disease severity, bulbar involvement, and adequacy of perioperative immunomodulatory management than to the surgical approach alone. Therefore, the timing, optimization, and continuation of IVIG, plasma exchange, corticosteroids, or other immunosuppressive therapies are likely critical.

Extended thymectomy was the predominant surgical strategy in reported cases, and the most common histological subtypes were World Health Organization type B1 and B2, similar to thymomas associated with MG (Table 1). Because SPS relapse has been reported after thymoma recurrence [18], achieving R0 resection is important for both neurological and oncological control. Extended thymectomy may also reduce residual thymic tissue that could harbor autoreactive T cells [26], potentially mitigating ongoing immune dysregulation.

In the present case, extended thymectomy resulted in sustained functional improvement despite persistently elevated anti-GAD antibody titers. Previous studies have shown poor correlation between antibody titers and clinical severity, supporting functional status as a more relevant indicator of treatment response [27]. The patient achieved independent ambulation and improved quality of life. However, weak anti-AChR antibody positivity indicates the need for long-term surveillance for MG development, SPS relapse, or thymoma recurrence [12, 14, 18].

This report is limited by its single-case design and the small number of comparable cases in the literature. Further accumulation of cases is needed to clarify optimal surgical timing, perioperative immunotherapy, and long-term neurological outcomes in thymoma-associated SPS.

## Conclusions

Extended thymectomy may contribute to sustained neurological and functional improvement in selected patients with thymoma-associated SPS, even when anti-GAD antibody titers remain elevated. Because SPS is vulnerable to perioperative stress, minimally invasive thymectomy may help reduce surgical invasiveness, but careful perioperative immunotherapy remains essential. Surgical treatment should be integrated into a multidisciplinary strategy that balances oncological radicality with individualized neurological stabilization.

## Data Availability

All data generated or analyzed during this study are included in this article. Further information is available from the corresponding author on reasonable request.

## Abbreviations

AChR: Anti-acetylcholine receptor
GABA: γ-Aminobutyric acid
GAD: Glutamic acid decarboxylase
IVIG: Intravenous immunoglobulin
MG: Myasthenia gravis
SPS: Stiff-person syndrome
WHO: World Health Organization

## References

[1] Dalakas MC. Stiff-person syndrome and GAD antibody–spectrum disorders: GABAergic neuronal excitability, immunopathogenesis, and update on antibody therapies. Neurotherapeutics. 2022;19:832–47. 35084720 10.1007/s13311-022-01188-w PMC9294130

[2] Murinson BB, Guarnaccia JB. Stiff-person syndrome with amphiphysin antibodies. Neurology. 2008;71:1955–8. 18971449 10.1212/01.wnl.0000327342.58936.e0 PMC2676978

[3] Smith SR, Fu JB. Paraneoplastic stiff-person syndrome: inpatient rehabilitation outcomes of a rare disease from two cancer rehabilitation programmes. J Rehabil Med. 2016;48:639–42. 27157044 10.2340/16501977-2089

[4] Solimena M, Folli F, Aparisi R, Pozza G, De Camilli P. Autoantibodies to GABAergic neurons and pancreatic beta cells in stiff-man syndrome. N Engl J Med. 1990;322:1555–60. 2135382 10.1056/NEJM199005313222202

[5] Matsui N, Tanaka K, Izumi Y. Stiff-person syndrome. Brain Nerve. 2023;75:749–54. 37287358 10.11477/mf.1416202410

[6] McKeon A, Robinson MT, McEvoy KM, Matsumoto JY, Lennon VA, Ahlskog JE, et al. Stiff-man syndrome and variants: clinical course, treatments, and outcomes. Arch Neurol. 2012;69:230–8. 22332190 10.1001/archneurol.2011.991

[7] Lenglet T, Honnorat J, Attarian S. Systematic review of immune and symptomatic treatments for stiff-person syndrome. Eur J Neurol. 2025;32:e70435. 41273190 10.1111/ene.70435 PMC12639332

[8] Vernino S, Lennon VA. Autoantibody profiles and neurological correlations of thymoma. Clin Cancer Res. 2004;10:7270–5. 15534101 10.1158/1078-0432.CCR-04-0735

[9] Veira J, Manes RP, Cormier NR. The anesthetic management of a patient with stiff person syndrome undergoing ambulatory endoscopic sinus surgery: A case report. JCA Adv. 2025;2:100157.

[10] Okado S, Mizuno T, Kawasumi Y, Kadomatsu Y, Chen-Yoshikawa TF. A case of myasthenia gravis and stiff-person syndrome that improved after extended thymectomy. Respirol Case Rep. 2025;13:e70149. 40099029 10.1002/rcr2.70149 PMC11913529

[11] Tanaka H, Matsumura A, Okumura M, Kitaguchi M, Yamamoto S, Iuchi K. Stiff-man syndrome with thymoma. Ann Thorac Surg. 2005;80:739–41. 16039251 10.1016/j.athoracsur.2004.02.076

[12] Nicholas AP, Chatterjee A, Arnold MM, Claussen GC, Zorn GL Jr, Oh SJ. Stiff-person syndrome associated with thymoma and subsequent myasthenia gravis. Muscle Nerve. 1997;20:493–8. 9121508 10.1002/(sici)1097-4598(199704)20:4<493::aid-mus13>3.0.co;2-#

[13] Hagiwara H, Enomoto-Nakatani S, Sakai K, Ugawa Y, Kusunoki S, Kanazawa I. Stiff-person syndrome associated with invasive thymoma: a case report. J Neurol Sci. 2001;193:59–62. 11718752 10.1016/s0022-510x(01)00602-5

[14] Thomas S, Critchley P, Lawden M, Farooq S, Thomas A, Proudlock FA, et al. Stiff-person syndrome with eye movement abnormality, myasthenia gravis, and thymoma. J Neurol Neurosurg Psychiatry. 2005;76:141–2. 15608018 10.1136/jnnp.2004.036558 PMC1739318

[15] Iwata T, Inoue K, Mizuguchi S, Morita R, Tsukioka T, Suehiro S. Thymectomy for paraneoplastic stiff-person syndrome associated with invasive thymoma. J Thorac Cardiovasc Surg. 2006;132:196–7. 16798340 10.1016/j.jtcvs.2006.03.030

[16] Essalmi L, Meaux-Ruault N, Hafsaoui C, Gil H, Curlier E, Dupond JL. Stiff-person syndrome associated with thymoma: efficacy of thymectomy. Rev Med Interne. 2007;28:627–30. 17624641 10.1016/j.revmed.2007.05.010

[17] Dupond JL, Essalmi L, Gil H, Meaux-Ruault N, Hafsaoui C. Rituximab treatment of stiff-person syndrome in a patient with thymoma, diabetes mellitus, and autoimmune thyroiditis. J Clin Neurosci. 2010;17:389–91. 20071184 10.1016/j.jocn.2009.06.015

[18] Kobayashi R, Kaji M, Horiuchi S, Miyahara N, Hino Y, Suemasu K. Recurrent thymoma with stiff-person syndrome and pure red blood cell aplasia. Ann Thorac Surg. 2014;97:1802–4. 24792276 10.1016/j.athoracsur.2013.07.103

[19] Morise S, Nakamura M, Morita JI, Miyake K, Kunieda T, Kaneko S, et al. Thymoma-associated progressive encephalomyelitis with rigidity and myoclonus (PERM) with myasthenia gravis. Intern Med. 2017;56:1733–7. 28674368 10.2169/internalmedicine.56.7979 PMC5519481

[20] Mehta MP, Sokol LL. The case of a 30-year-old man with subacute gait instability, weakness, and muscle spasms. Ann Clin Transl Neurol. 2020;7:2535–7. 33174672 10.1002/acn3.51181 PMC7732238

[21] Sasaki A, Kato T, Ujiie H, Wakasa S, Otake S, Kikuchi K, et al. Thymoma-related stiff-person syndrome successfully treated by surgery. Ann Thorac Cardiovasc Surg. 2022;28:448–52. 34275989 10.5761/atcs.cr.21-00052 PMC9763711

[22] Perri M, Pellegrini D, Uribe Roca C, Gonzalez F, Buero A, Chimondeguy D, et al. Stiff-person syndrome associated with thymoma. Medicina (B Aires). 2023;83:626–30. 37582138

[23] Sonokawa T, Shizukawa H, Ichihara S, Muraoka S, Usuda J, Tanaka K. Case report: stiff person syndrome with thymoma. Jpn J Lung Cancer. 2019;59:360–5.

[24] Sumiya R, Banno T, Ueno H, Hirayama S, Suzuki K. Efficacy of surgical treatment for thymoma-related stiff person syndrome. Ann Thorac Surg Short Rep. 2024;2:540–3. 39790377 10.1016/j.atssr.2024.01.004 PMC11708581

[25] Fujii Y. Thymus, thymoma, and myasthenia gravis. Surg Today. 2013;43:461–6. 22948665 10.1007/s00595-012-0318-2

[26] Rakocevic G, Raju R, Dalakas MC. Anti–glutamic acid decarboxylase antibodies in the serum and cerebrospinal fluid of patients with stiff-person syndrome: correlation with clinical severity. Arch Neurol. 2004;61:902–4. 15210528 10.1001/archneur.61.6.902

[27] Bose S, Jacob S. Stiff-person syndrome. Pract Neurol. 2025;25:6–17. 39222980 10.1136/pn-2023-003974

